# From the Frontline to the Homefront: The Experience of Israeli Veterans

**DOI:** 10.3389/fpsyt.2020.589391

**Published:** 2020-10-28

**Authors:** Zahava Solomon

**Affiliations:** ^1^Bob Shapell School of Social Work, Tel-Aviv University, Tel-Aviv, Israel; ^2^I-Core Research Center for Mass Trauma, Tel-Aviv University, Tel-Aviv, Israel

**Keywords:** veteran, war, PTSD—posttraumatic stress disorder, trauma, stress, psychopathology

## Abstract

In 1948, the state of Israel was created as a homeland for the Jewish people after 2,000 years of persecution and deportations in the diaspora. During the past 72 years, its inhabitants have experienced several wars and numerous terrorist attacks. Therefore, the issue of trauma goes beyond academic study, it is part of daily life. These circumstances have, unfortunately, turned Israel into a natural stress laboratory, which has enabled the systematic research of the biopsychosocial effects of traumatic stress on soldiers and civilians. This article reviews the findings of a series of studies that examine (a) the short- and long-term mental health effects of war on combat veterans; (b) the effects of repeated exposure to war on veterans; (c) trajectories of PTSD; and, specifically, (d) reactivation and (e) delayed-onset PTSD. We present the findings of two decades of systematic trauma research, which have followed the ongoing psychopathological effect of war on veterans. In understanding the ripple effects of trauma, it can be seen that veterans do not leave the events of the war behind once they are home; rather, it is with them wherever they go. Consequently, the trauma has a ripple effect that may carry over to veterans' spouses and offspring. The multiple manifestations and trajectories of both acute and chronic trauma will be presented. Clinical ramifications and implications will also be discussed.

## Introduction

War is one of the most devastating human constructs. There are the visible results of war, such as the loss of life and the destruction of property and culture (1). However, there is also the invisible toll of psychological damage (1, 2). In the time of war, combatants not only face ongoing danger while in the active field of duty; they also witness the injury and death of their fellow soldiers, commanders, and enemies as well as innocent civilians. Moreover, combatants are not only passive bystanders, they are also perpetrators who cause death, injury, and destruction, thereby violating the ethical standards and values commonly held during peacetime (3). They struggle with loneliness, isolation, and forced separation from their loved ones alongside a lack of their physical needs being met in regard to food, drink, and sleep. Adding to the already massive stress is the unpredictability of modern warfare, including weapons of mass destruction and guerrilla warfare, which makes it difficult to predict where and when the next attack could occur (1). While most soldiers are able to adequately cope with these stressors, others become so overwhelmed by combat stress that their psychological defenses become exhausted, causing psychological breakdown.

The stress of war may have immediate, long-term, and delayed psychiatric consequences [e.g., (4–6)]. Psychological disorders resulting from war are often acknowledged either at the outbreak or immediately following the end of the war, frequently with a lack of ongoing, systematic long-term follow-up for those who are impacted (1). Hence, much of the knowledge in this field is rather fragmented and, in many cases, knowledge that has been previously gathered does not get passed on in the event of another war (7). Moreover, the structure and diverse policies of various armies and Ministries of Defense throughout the world do not allow for systematic longitudinal studies of traumatized combatants. The unique circumstances in Israel, however, permit and even support the systematic research of traumatized veterans. Specifically, Israel has a mandatory 3-year military service for all able-bodied young adults and continuous reserve duty for men until the age of 55. Furthermore, the country is small; all Israeli wars are fought on Israeli land and its borders and, it is significant to note the close link between the Israel Defense Forces (IDF) and the Ministry of Defense. Capitalizing on these characteristics, we have initiated and conducted longitudinal studies spanning two decades of several cohorts of traumatized Israeli veterans [for details see (2, 8)].

These Israeli studies addressed many questions that had been left unanswered by previous research, as outlined by Solomon (1): What are the frequency and duration of psychiatric breakdown on the battlefield? What are the characteristic manifestations of combat-induced psychopathology? Does recurrent exposure to combat weaken soldiers or lead to greater resilience? How can we explain the many recovered combat stress reaction (CSR) casualties whose traumas are reactivated after a long asymptomatic period? Is delayed posttraumatic stress disorder (PTSD) onset a valid phenomenon? And if so what explains its onset a long time after exposure to trauma? This review article summarizes some of our studies' findings, casting light on these important questions.

## Combat Stress Reaction

Combat stress reaction is the most common and conspicuous immediate psychological reaction to war, also known as shell shock, combat exhaustion, or war neurosis (2). CSR occurs when a combatant is unable to cope effectively with the threatening stimuli they are experiencing while partaking in active duty. Its diagnostic parameters include a wide range of symptoms, which may wax and wane rapidly, including restlessness, psychometric deficiencies, withdrawal from others, increased sympathetic activity, confusion, and paranoia, among others (9). The clinical picture of CSR is seen as polymorphic and labile. The most telling symptom of CSR is when the soldier is no longer able to engage in combat, thereby acting in a way that endangers the lives of his or her fellow combatants as well as him or herself (10). Unlike most psychiatric disorders, however, CSR does not allow for a clearly defined pattern of symptoms; rather, it is defined in a more general and functional way. Therefore, we considered it important to assess its typical clinical picture. We conducted a content analysis of a random sample of 104 medical charts of CSR casualties of the 1982 Lebanon War (11) and identified the following symptom clusters: psychic numbing and dissociation; anxiety symptoms, guilt about a failure to function, sense of helplessness and loneliness; and psychosomatic and psychotic-like symptoms (disorientation). Taken together, this classification of CSR is similar to that of Grinker and Spiegel (4). Although their study took place under a different cultural background and following World War II (WWII), it underscores the universality of CSR manifestations. Similar to earlier reports from World War I and WWII, the prevalence of CSR was also found to present in diverse ways during various battles and was directly related to the number of physical causalities in each battle (2).

## Is CSR Short Lived and Transient?

CSR has often been described in the international military mental health literature as a short-lived phenomenon. It has been argued that CSR is a rational reaction to an irrational situation (1) where once the distressed soldier is removed from the stressors of war, they will regain their equilibrium and functionality (10, 12). To the best of my knowledge, the majority of large-scale prospective studies of CSR have been conducted in Israel involving combatants from the 1973 Yom Kippur War [e.g., (13)] and the 1982 Lebanon War [e.g., (2, 8)]. In particular, in the Yom Kippur study, CSR casualties were identified as a high-risk group for PTSD and other psychiatric and somatic co-morbidities (13).

A more elaborated series of studies commenced during the 1982 Lebanon War as the IDF began a longitudinal research project that followed 382 CSR casualties and 334 un-afflicted control soldiers, who were carefully matched to the CSR group in sociodemographic and military characteristics [e.g., (1, 14)]. In this 20-year longitudinal study, we followed the effects of combat, namely, the resulting psychological and somatic conditions for both groups. Assessments were conducted 1, 2, 3, and 20 years after the 1982 war. Our main aim was to examine the effects of combat stress, as manifested in CSR, and to understand whether it begins and ends on the battlefield or if it continues after the war is over. We found that, for a large number of Israeli veterans, their war trauma left ongoing and disruptive sequelae. Rates of PTSD were rather high among the identified CSR combatants during the 20-year study: 1 year postwar, 54%; 2 years, 47%; 3 years, 38%; and 20 years after the war, 27%. In other words, for a substantial proportion of veterans who had a psychological breakdown on the battlefield, the war was not over when the shooting stopped. This means that for many CSR casualties, the initial breakdown on the frontline marked the beginning of a lifelong struggle with the psychopathological effects of war.

Those who experienced CSR during combat were found to have a higher likelihood to develop PTSD throughout the study. Interestingly, the long-term pathogenic effects of CSR were not only reflected in higher PTSD rates but also in its severity. Throughout the 20-year follow-up, we found that veterans who had CSR also suffered from more severe PTSD than comparable combat veterans who did not suffer from CSR (2, 8, 15). These findings speak to the predictability of CSR in later PTSD (8).

PTSD was not the only detrimental outcome found for the CSR veterans (14). The findings also revealed that these veterans endured significantly greater psychiatric symptomatology, distress, social functioning difficulties (15), health-related problems (16), accelerated aging (17), and earlier all-cause mortality (18) than those in the non-CSR group.

Studies conducted more recently examined the effects of conflicts in Iraq, Syria, and Afghanistan. Veterans in Iraq [e.g., (19, 20)] who were assessed psychiatrically and evacuated from the frontlines received diagnoses of acute stress disorder (ASD) or acute stress reaction (ASR). Unfortunately, as these studies were after the veterans' return home it is likely that many others were overlooked who had not been evacuated and, furthermore, none used the CSR paradigm. Other systematic recent studies such as these, for example, carried out by the Danish Veterans Center, assessed PTSD symptoms rather than ASD [e.g., (21)].

The unique series of Israeli longitudinal studies of CSR casualties attests to the entrenched and enduring effects of CSR. Our findings refute the long-held notion that CSR is merely a normal yet short-lived episode that subsides once the immediate danger that combat trauma entails is lifted. Importantly, while CSR can be seen as a specific case of ASR, the two differ in that CSR is diagnosed using functional rather than symptomatic criteria (14). In other words, the psychological breakdown on the battlefield reflects the soldier's lack of restraining their fear response and returning to a stable condition (8).

CSR, as assessed in our studies, appears to be a robust marker of PTSD. Indeed, our studies indicated that, for many, CSR marks the beginning of a course of lifelong chronic PTSD and posttraumatic decline, reflected also in poor professional functioning and impaired social and familial relations (2, 15). It, therefore, transpires that CSR is not a temporary disruption, but rather a considerable and lifelong vulnerability. The experience of CSR is the quintessential moment wherein a combatant begins to feel vulnerable and helpless as they lose their senses of safety and ability (8). Another consideration is that the veterans who experienced CSR, followed by severe PTSD, may have been more at risk prior to battle than those who were not found to have CSR. At the same time, however, the CSR and comparison groups did not significantly differ in any pre-military screening, including physical and psychiatric measures (8). Although the evidence does not rule out the prior suggested possibilities, it is more plausible that CSR is the beginning of the path to the development of PTSD. Regardless of causation, based on the findings it is of the utmost importance for veterans who have experienced CSR to receive ongoing clinical support.

## Effects of Repeated Exposure To Combat

The circumstances in Israel, unfortunately, have led to the necessity and ability to examine the effects of repeated exposure to combat (1). As there are few countries in the world where soldiers must continue to fight in multiple wars, there is limited knowledge on combat stress. The theoretical basis in predicting CSR derives from general psychological and somatic studies, which suggest three different perspectives. First, the vulnerability perspective [e.g., (22)] whereby exposure to stressful events on a repeated basis is a risk factor, as it depletes coping resources, increasing the individual's vulnerability. Second, the stress inoculation perspective [e.g., (23)] suggests that exposure to repeated stress is protective due to the establishment of effective coping strategies and adaptation to the situation. The third view, the stress resolution hypothesis, proposes that it is the way that a person copes with the stress that is imperative not, *per se*, the exposure to stress. Block and Zautra (24) explain further that when one is able to cope successfully with stress, they develop a sense of well-being that encourages further healthy coping, conversely, unsuccessfully coping with stress may increase one's sense of distress that further undermines their ability to cope.

To assess the effects of repeated exposure to combat, we presented the Israeli combatant participants from the 1982 Lebanon War with a list of seven Israeli wars. We then asked them to indicate which wars they were combatants in and if they had experienced CSR in those wars (2, 25). We found that the highest rates (66%) of CSR were observed among combatants who had a previous diagnosis of CSR, the lowest rates (44%) were found among those who had previously been in combat and not experienced CSR; a rate of 57% was found for those who had no previous combat experience (1). Therefore, these findings suggest that coping successfully with past stress does indeed lead soldiers to, again, cope successfully in any following combat. However, it should also be noted that soldiers with previous CSR fared worse than those without any preceding battle experience. Previous experience of CSR does not signify that a soldier will definitively experience it again if put in active duty, although, according to the results, it does leave that soldier more vulnerable the second time around. Those who do return to the battlefield are few, as they were both deemed fit to return to active military duty and expressed personal motivation to do so (1). Thus, if this highly selected group of veterans display increased vulnerability, the other CSR casualties are likely even more vulnerable.

When examining the results, it can be seen that partaking in combat over numerous wars has a deleterious effect. Among veterans with a history of CSR, the risk of subsequent CSR increases according to the number of previous wars: 57% after one war, 67% after two, and 83% after three. Among seasoned combat veterans without a history of CSR in the 1982 Lebanon War, 50% of those who fought in one previous war, 44% who fought in three wars, and 33% who fought in two wars displayed higher CSR rates (1). These findings suggest that combat scars combatants and weakens their resilience. Furthermore, despite the suggestion that previous successful coping will lead to stress inoculation, in due course repeated stress exposure will cause the fall of the strongest of soldiers.

## Trajectories of PTSD

The course of PTSD tends to fluctuate resulting in multiple trajectories that vary in severity and duration. The literature concerning these trajectories over time include groundbreaking large-scale retrospective American epidemiological studies [e.g., (26)], later follow-ups of Vietnam veterans [e.g., (27)], systematic prospective Danish and Dutch studies [e.g., (21, 28)], and several longitudinal studies of civilians [e.g., (29, 30)]. After the inclusion of ASD in the DSM-4, there was an increasing interest, resulting in several studies [e.g., (31, 32)]. However, the follow-up of these studies has been fairly short-term, only covering limited periods of time. Hence, the long-term course of combat induced PTSD and psychopathology requires further scientific validation.

The 20-year prospective study of Israeli veterans from the 1982 Lebanon War in relation to antecedent CSR revealed fluctuations of PTSD in both groups (8, 33). This fluctuating course was characterized by relapses/reactivations and remissions in both of the study groups. Delayed onset, defined in this study by an appearance of PTSD at any point from the measurement at the first year postwar without any previous symptoms, was reported by 23.8% of the comparison group and 16.1% of the CSR group. Specifically, 8.4% of the comparison group and 4.6% of the CSR veterans displayed PTSD only at the 20-year postwar mark, 1.2% of the comparison group and 0.8% of the CSR group at the 3-year measurement, and 3.6% of the comparison group and 2.3% of CSR group at the 2-year measurement (8).

Regarding the remissions observed in both groups (8), 7.6% of the veterans in the CSR group displayed one occurrence of remission followed by another event of PTSD, 4.6% had two remissions followed by PTSD, and 3.8% had three remissions followed by PTSD. Complete remission was seen for 3.8%; 16.8% had remission twice and 23.7% once. For the comparison group, 13.3% exhibited PTSD once, 8.4% twice, and 2.4% three times. Complete remission was seen for 3.6%; 3.6% PTSD had two remissions and, 3.6% had one remission.

## Reactivation of PTSD

Of the various PTSD trajectories, two deserve special attention: reactivation and delayed onset. Reactivation is defined as PTSD that is triggered again after exposure to a subsequent stressor, often similar to the original trauma. Reactivation of stress reactions has been observed in survivors of various traumas as the current stressors reactivate anniversary reactions (34). For example, this has been found among widows who, when reminded of the loss of their spouses, experienced a reactivation of their grief [e.g., (35)]. Rape victims have also been found to have a reactivation of their trauma response upon reminders of the initial trauma (36). American WWII veterans as well as Holocaust survivors have been found to report a reactivation of wartime trauma responses coinciding with aging-related losses (37, 38). In addition, veterans from the Vietnam War have reported reactivation of their symptoms when they visited war memorials or public events that reminded them of their experience during the war [e.g., (39)].

My interest in this phenomenon began after reading the medical files of the traumatized veterans from the 1982 Lebanon War. I was struck that many veterans repeatedly spoke about their combat experiences in previous wars. To better understand this phenomena, a team of four mental health professionals carefully reviewed 35 such cases (40). We found considerable variability in relation to behavioral and functional impairment ranging from very mild to extreme.

### Uncomplicated Reactivation (23%)

These veterans appeared to have experienced a complete recovery from their previous stress reaction and did not seem to endure any further symptoms while not on the battlefield. Their current stress reactions were generally caused after an incident that triggered their prior initial traumatic experience (1).

The other participants in this study fell under the category of exacerbated PTSD. In these cases, the previous war trauma left more of an impact as the veterans continually experienced PTSD symptoms at varying degrees. For example, PTSD symptoms were found to increase while on reserve duty and also during the conscription for the 1982 Lebanon War out of anticipation of having to return to war. Additionally, these veterans were also vulnerable to experience reactivated PTSD as a result of unconnected events that did not directly threaten their immediate safety. We subdivided exacerbated PTSD into three groups (1):

### Heightened Vulnerability (51%)

These veterans experienced mild, diffused PTSD symptoms, which did not impede daily their functioning, and increased sensitivity to military stimuli. Their residual distress became fully developed PTSD after facing a direct military threat during the Lebanon War, usually not unlike the event that precipitated the original breakdown.

### Moderate Generalized Vulnerability (9%)

These veterans displayed a moderate generalized sensitivity both in their civilian lives and during reserve duty. They suffered from some residual PTSD symptoms (e.g., irritability, sleep problems, uncontrollable outbursts of anger) that somewhat impaired their functioning. When these veterans returned to the war front during the 1982 Lebanon War, they quickly developed a stress reaction in relation to rather minor military related events, with many being discharged prior to partaking in active combat.

### Severe Generalized Sensitivity (19%)

A minority of veterans suffered from PTSD symptoms throughout the time between wars. For these veterans, the arrival of their conscription order for the 1982 Lebanon War resulted in an immediate and debilitating stress reaction. Many did not even engage in combat or reach the frontline before experiencing their second psychological breakdown (41). It should be noted that all of the veterans in our study who had reactivated or exacerbated PTSD after the 1982 Lebanon War made a considerable effort to function during the 9 years between the two wars and were rather successful. Most had married and were gainfully employed, with some prospering professionally. None of the veterans had been hospitalized due to mental health issues, and all continued to participate in reserve duties despite their intensifying symptoms when faced with military stimuli. The second wave of stress reaction shed light on the psychological damage sustained during the first breakdown and further escalated it. In general, the second episode was more intense and debilitating than the first (42).

## Delayed-Onset PTSD

The psychological wounds of combat erupt in the form of PTSD either during or after exposure to traumatic events. The DSM-5 (43) considers delayed-onset PTSD (DPTSD) to occur when PTSD is first evident 6 months or subsequently posttrauma. Despite being acknowledged by the DSM, the validity of the existence of DPTSD has been under debate by both medical and legal professionals. This diagnosis comes into question particularly due to the possible financial benefits a veteran may receive if diagnosed with PTSD (44). Moreover, there is also the question of whether it is truly DPTSD or malingering PTSD (45). Others assert that it is the treatment or diagnosis of symptoms that is deferred rather than the actual delayed onset of the PTSD itself [e.g., (46)], meaning that PTSD may have been present and active without being properly diagnosed (47). Despite the considerable skepticism and questioning of the validity of DPTSD, many studies have supported its existence. DPTSD has been observed among survivors of various traumatic events including motor vehicle accidents (48), natural disasters (49), incest (50), and combat (51). Moreover, credible findings were reported in a meta-analysis of prospective DPTSD studies (52), providing further support for the existence of this phenomenon.

In the literature, there are inconsistent findings regarding DPTSD prevalence, process of onset, relative severity, and the relationship between acute reactions and DPTSD. For instance, estimates of DPTSD prevalence have been reported to range from 0% (23) to more than 60% (53). Therefore, we aimed to examine the prevalence of DPTSD in our prospective longitudinal study, including several follow-up measurements (44). A second remaining question relates to the timing of the appearance of DPTSD, namely, whether it occurs only after a lack of symptoms or after a culmination of ongoing symptoms (47). We, therefore, set out to study whether DPTSD symptoms occurred only after an asymptomatic period or if it surfaced after an increase in residual subclinical symptoms over time. Also left unanswered is the question of DPTSD symptom severity in relation to symptoms of PTSD that is not delayed. While some studies have failed to establish a relation between the time of PTSD onset and extent of psychopathology [e.g., (54)], others have found DPTSD to be less severe than immediate PTSD (55, 56). Therefore, assessing DPTSD symptom severity is an additional aim in our study. Furthermore, it is unclear regarding possible associations between ASD and DPTSD. To the best of my knowledge, only one study has examined this question, however, a connection was not found (56). Given the profound differences between ASD and CSR discussed above, we set out to examine the association between antecedent CSR and DPTSD.

We assessed DPTSD prevalence in Israeli veterans from the 1982 Lebanon War, both with and without antecedent CSR, with measurements at 1, 2, and 20 years after the war using two methods (44). We found that for a significant number of veterans, there were reports of DPTSD up to 20 years after the end of the war. First, when we examined all of the participants together, 16.5% were found to have DPTSD, which is higher than previously found in a majority of studies [e.g., (57)], although others have reported similar rates [e.g., (58)]. Second, in comparing only the veterans with PTSD, a higher rate of 27% endorsed DPTSD. In other previous studies, similar calculations rendered rates ranging from zero to 68% (47). Nonetheless, our finding of 27% corresponds with that of Smid et al.'s (52) meta-analysis of prospective DPTSD studies, which reported an average of 25%. Additionally, this finding gives strength to clinical observations of DPTSD that have noted endorsements of PTSD arising decades after the initial trauma and, furthermore, that delays of longer or shorter periods of time are equally likely (59).

Our comparatively high DPTSD rate also is in line with Prigerson et al. (60) and a recent short-term prospective study of Danish veterans (21) who observed that DPTSD is common in particular among war trauma survivors. There are several possible explanations for this prevalence. First, while on the battlefield, it is imperative that combatants are highly alert and functioning at their highest level. Conversely, after their return home they are able to be less aware and vigilant. In this way, soldiers will often only experience and convey feelings of distress sometime after the combat (61). A second consideration is that of the stigma related to PTSD, particularly in Israel, where there are many wars and soldiers are expected to be brave and not negatively impacted by events on the battlefield. Hence, soldiers may try to conceal, inhibit, or delay any symptoms of emotional distress (44). A third explanation, relating to the high rate of DPTSD found in our study, is that Israel could be considered a “stress lab,” meaning that those who live here experience an ongoing exposure to war and terror, which, in turn, could trigger past trauma, regardless of the amount of time that has passed. DPTSD has been found to be linked to external stimuli; this implies that circumstances or events that may resemble the original trauma thereby provoke its memory [e.g., (50)]. Living under these conditions, there are many occasions where an earlier war trauma could be triggered, despite the amount of time that has elapsed.

The occurrence of DPTSD 20 years postwar found in our study is consistent with previous research that has reported prolonged delays of PTSD onset during mid-life and old age (59, 62). These stages of life come with more time to reflect and reminisce on one's life up until this point. This includes recalling past traumatic events (44) and inherent losses and endings, such as retirement, illness, and the passing of loved ones, which could be especially upsetting for trauma survivors (38), as well as reduced psychosocial resources (e.g., activities, social connections), health, and status (51, 63, 64). It also transpires that both cognitive (60) and biological (65) factors that are associated with old age are associated with DPTSD. Indeed, there is a growing understanding that DPTSD and processes related to aging could be linked (59, 66).

Possible relations between CSR and DPTSD were also explored in our study. The findings showed that experiencing a mental health breakdown on the battlefield was associated with a shorter time period until the appearance of DPTSD (44). As noted in a review of the ASD literature (14), CSR has been found to be an important predictor of ensuing and reactivated PTSD. Our findings suggest that the presence of CSR may go beyond the prediction of PTSD and also predict when PTSD may appear.

Past studies on the intensity of DPTSD symptomology have reported varied results [e.g., (54, 55, 67)]. However, the results from our study distinctly show that with longer delays of DPTSD there was lower psychopathology. The group that was found to have DPTSD 1-year postwar was the most vulnerable, followed by the 2- and 20-year postwar onset groups, who were similar in the majority of the psychopathology measures. The no prior PTSD group had the lowest psychopathology levels, as predicted. Therefore, it could be suggested that the amount of time in the delay is a vital factor in predicting psychopathology severity. Namely, up until 1 year, there could be an increase in severity, with later delays not having as significant an impact on psychopathology.

In relation to these findings, it could be suggested that over time there is a decline in the impact of the combat and, therefore, when it is triggered after a longer period of time, it is less severe. It could also be that the PTSD was delayed as a result of more resilient coping abilities immediately following the war, which aligns with Smid et al.'s (52) findings that lower accumulative symptoms of PTSD were related to increased reports of DPTSD. Hence, it could be deduced that the aspects that played a part in the delay of PTSD for some veterans could encourage resilience for others who, thereby, do not develop PTSD. It is also possible that the resilience of these veterans is amplified by their ability to gain resources, such as supportive social networks and gainful employment during times where they are asymptomatic.

What can account for the onset of DPTSD decades postwar? This delayed onset could be due to two factors (44). First, the 20-year postwar assessment of this study took place during a particularly intense and volatile conflict between Israel and the Palestinians (Intifada Al Aqsa). During this time, Israeli civilians faced almost daily terrorist attacks, which was also found to be implicated in posttraumatic distress (68) and, hence, these events could justifiably trigger DPTSD. Second, in regard to the 20-year delayed PTSD, the delay could be due to the stage of life of the veterans who were entering into their mid-life, with its abovementioned losses and reflections.

In general, it was found that veterans with CSR experienced a higher severity of DPTSD than those without CSR (44). This corresponds to previous studies that have reported individuals with ASD (69) and CSR (15) to be at a higher risk for subsequent psychopathology. It is also in line with the “vulnerability perspective,” which suggests that experiencing a primary trauma may compromise effective coping strategies in later events of distress (70). Additionally, stress sensitization, whereby exposure to severe stress could increase responsiveness during future stressful events, has been proposed as a possible underlying mechanism in DPTSD in several studies (71, 72) and has also received empirical support (73), meaning that CSR could contribute to this sensitivity.

## Does DPTSD Erupt After A Truly Asymptomatic Period?

We found that, in general, DPTSD occurred following residual PTSD symptoms. Specifically, among the 2-year postwar and 20-year postwar DPTSD onsets as well as the no prior PTSD group there was evidence of an increase in existing symptoms before the onset of PTSD (44). This is supported by Smid et al.'s (52) findings that DPTSD frequently results from an increase in subthreshold PTSD symptoms as well as research regarding DPTSD in non-combat related traumas (47, 59, 74). One explanation that has been recently suggested as a model for DPTSD is “fear incubation” (75), whereby fear conditioning results in increasing fear and anxiety over time. Studies of rats have reported that, post-fear training, conditioned fear responses were markedly higher after 31 and 61 days than after 2 or 15 days (76). As such, fear incubation replicated a heightened response to trauma triggers over time, similar to that seen in DPTSD. In another animal PTSD study (77), after classical conditioning, an amplified response was found after a similar period of delay. This type of response is known as “conditioning-specific reflex modification” (CRM) and has been found to be relevant for specific aspects of PTSD (78). Their results showed a pattern similar to that of PTSD symptoms that have a delayed presentation as, when they appear, they continue and worsen over time. In our study, DPTSD emerged among a small number of participants after an asymptomatic period. Although some are of the opinion that this is an unlikely manifestation of PTSD [e.g., (79)], this pattern of DPTSD has been previously reported by others [e.g., (80)], particularly in case studies (81).

## Limitations

This series of studies has several methodological limitations to consider. First, self-report measures were used that, although often found in trauma studies, may have a risk of biased reporting. Another limitation is related to the timing of the measurements. Due to the gap between the assessments, there is a lack of data regarding the veterans' potential PTSD between the waves of measurement. Furthermore, attrition may have had an impact in this longitudinal study. Although sophisticated missing data strategies were employed, it must be acknowledged that participant drop-off may have unknowingly affected our results. The study was conducted among Israeli combat veterans who fought in the same war and thus share a similar social and cultural characteristic. Generalization from these results to other populations, in other times and cultures, should be undertaken cautiously. Finally, this review is not systematic and there is no meta-analytic data and so the validity of the findings is somewhat limited.

Despite these limitations, the present series of studies make important contributions to the literature on veterans' mental health. The studies are based on a prospective design that commenced on the battlefield and continued over a 20-year follow-up period. Following veterans over such an expanse of time allows for a unique opportunity in the study of prevalence, correlations, and the clinical picture of combat-induced PTSD.

## Clinical Implications

These studies entail important theoretical implications. First, our results propose that the deleterious effects of combat are deep and enduring. The findings also highlight the significance of CSR in predicting subsequent PTSD and debilitating comorbidities. The transition from CSR to PTSD is an evolving process. It is seen as a window of opportunity for prevention of PTSD. Previously, this disorder crystallized and became entrenched and debilitating. In fact, we capitalized on our longitudinal studies of the CSR casualties of the 1982 Lebanon War and assessed the effectiveness of Front Line treatment and found that 1 year and 20 years after the war, CSR casualties who were treated using this modality suffered from much lower rates of PTSD and led more productive and stress-free lives (82, 83). Interventions for the acute phase should be adopted to impede and halt the progression from the acute to the chronic phase.

The longitudinal design indicates that the course of PTSD has considerable variability, with numerous trajectories, varying in duration and severity. Our findings also confirm the actuality of PTSD reactivation and DPTSD. The rates reported in this study unquestionably demonstrate that DPTSD is not a trivial or non-existent phenomenon. Therefore, it should be acknowledged as being one form that PTSD can take while having a unique clinical profile. Finally, the results also indicate that the presence of CSR is a significant risk factor, not only for PTSD but also for those who develop PTSD after a shorter delay.

There are also important practical implications to consider. PTSD is the only known disorder to specifically result from exposure to a traumatic event, which often involves medical, legal, and political implications. Traumatized veterans who are sent to war by their home countries must have and deserve long-term support, monitoring, and professional attention, specifically due to the labile nature of the PTSD course and particularly concerning reactivated PTSD and DPTSD.

With regard to DPTSD, mental health professionals are encouraged to closely follow their patients' emerging symptoms, whether or not the clinical threshold for PTSD has been reached. Also, the knowledge that psychopathology varies among those with PTSD could be imperative for therapists. Moreover, the timing of the PTSD onset must also be established, as it could have an effect on decisions regarding treatment. Additionally, as PTSD consists of unique subtypes, therapists should be encouraged to design interventions specifically for veterans with PTSD related conditions [e.g., (84)]. Finally, and most importantly, the plight of traumatized veterans across the globe stems from man's proclivity for aggression and the tendency of nations to settle political conflicts *via* wars. The lifelong suffering of traumatized veterans and their families, *via* secondary traumatization should be acknowledged and treated.

## Author Contributions

ZS made all of the contributions to the work and approved it for publication.

## Conflict of Interest

The author declares that the research was conducted in the absence of any commercial or financial relationships that could be construed as a potential conflict of interest. The reviewer YL declared a shared affiliation with the authors to the handling editor at time of review.
